# Cytotoxic and anticancer properties of the Malaysian mangrove pit viper (*Trimeresurus purpureomaculatus*) venom and its disintegrin (purpureomaculin)

**DOI:** 10.1590/1678-9199-JVATITD-2020-0013

**Published:** 2020-07-17

**Authors:** Choo Hock Tan, Jia Lee Liew, Suerialoasan Navanesan, Kae Shin Sim, Nget Hong Tan, Kae Yi Tan

**Affiliations:** 1 Department of Pharmacology, Faculty of Medicine, University of Malaya, Kuala Lumpur, Malaysia.; 2 Institute of Biological Sciences, Faculty of Science, University of Malaya, Kuala Lumpur, Malaysia.; 3 Department of Molecular Medicine, Faculty of Medicine, University of Malaya, Kuala Lumpur, Malaysia.

**Keywords:** Shore pit viper, Trimeresurus purpureomaculatus, Disintegrin, Selective cytotoxicity, Anti-neoplastic activity

## Abstract

**Background:**

The Asiatic pit vipers from the *Trimeresurus* complex are medically important venomous snakes. These pit vipers are often associated with snakebite that leads to fatal coagulopathy and tissue necrosis. The cytotoxic venoms of *Trimeresurus* spp.; however, hold great potential for the development of peptide-based anticancer drugs.

**Methods:**

This study investigated the cytotoxic effect of the venom from *Trimeresurus purpureomaculatus*, the mangrove pit viper (also known as shore pit viper) which is native in Malaysia, across a panel of human cancer cell lines from breast, lung, colon and prostate as well as the corresponding normal cell lines of each tissue.

**Results:**

The venom exhibited dose-dependent cytotoxic activities on all cell lines tested, with median inhibition concentrations (IC_50_) ranging from 0.42 to 6.98 µg/mL. The venom has a high selectivity index (SI = 14.54) on breast cancer cell line (MCF7), indicating that it is significantly more cytotoxic toward the cancer than to normal cell lines. Furthermore, the venom was fractionated using C_18_ reversed-phase high-performance liquid chromatography and the anticancer effect of each protein fraction was examined. Fraction 1 that contains a hydrophilic low molecular weight (approximately 7.5 kDa) protein was found to be the most cytotoxic and selective toward the breast cancer cell line (MCF7). The protein was identified using liquid chromatography-tandem mass spectrometry as a venom disintegrin, termed purpureomaculin in this study.

**Conclusion:**

Taken together, the findings revealed the potent and selective cytotoxicity of a disintegrin protein isolated from the Malaysian *T. purpureomaculatus* venom and suggested its anticancer potential in drug discovery.

## Background

Snake venom is widely regarded as an advanced “biochemical weapon” that exists in nature. Venomous snakes rely on venom for survival, where it is employed for predation (primary function), digestion and defense [1]. Rapid evolution drives the emergence of diverse pharmacologically active snake venom proteins that are adapted to target the normal physiology of prey by exerting various toxic effects [2]. The prey of venomous snakes consists in parts of small mammals such as rodents but may also include birds, lizards and amphibians [3, 4]. It is noteworthy that the mammalian system-targeting property of snake venom proteins is often specific and selective, thus making them a natural repertoire of therapeutic molecules [5, 6]. 

At present, advanced cancers that are fast-growing and capable of metastasizing is a major cause of mortality globally [7, 8]. In this context, snake venom proteins have the potential for drug discovery in line with the search for novel peptide-based anticancer agents with high efficacy and selectivity in targeting cancer cells [9, 10]. Notable examples typically involved venom proteins from vipers and pit vipers (family Viperidae*),* e.g. disintegrin from *Agkistrodon contortrix* venom [11], phospholipases A_2_ from *Cerastes cerastes* [12] and L-amino acid oxidases from *Bothrops* sp. [13]. 

A common pharmacological feature of the venoms of viperid snakes, including the Asian arboreal pit vipers from the *Trimeresurus* complex, is their ability to derange hemostasis and induce local tissue necrosis [14-16]. The tissue-necrotizing effect associated with *Trimeresurus* pit viper envenomation suggests cytotoxic activity in the venom that may be further explored for anticancer potential. A recent study with Thai *Trimeresurus purpureomaculatus* venom reported high cytotoxicity of the venom toward SHSY5Y cell line, and moderate cytotoxicity of BPP-related peptides, PLA_2_ and peptide-rich fraction of the venom [17]. Another publication reported that L-amino acid oxidase (LAAO, 55−60 kDa) from Malaysian *Trimeresurus purpureomaculatus* venom induced cytotoxicity in three colon cell lines (SW480, SW620 and CCD-18Co), but no selectivity was observed between the cancerous cell lines (SW480, SW620) and the non-cancerous, normal cell line (CCD-18Co) [18]. 

Our recent quantitative proteomic analysis of the Malaysian *T. purpureomaculatus* venom (MTP) revealed that LAAO constituted about 3% of the total venom proteins, while disintegrin, a compound with potential inhibitory effect on cancer cell growth, was present at a higher abundance in the venom (approximately 14%) [19]. The LAAO content reported previously for the Malaysian *T. purpureomaculatus* venom could not be compared as it was estimated with a qualitative method [20]. In a more recent study, relative protein abundances of 0.91% LAAO and 0.10% disintegrin were identified in the Thai *T. purpureomaculatus* venom [17]. 

Disintegrin is a much smaller (4-15 kDa, monomer or dimer) cysteine-rich and RGD-containing polypeptide that targets specifically on integrin - a ubiquitously expressed cell surface receptor that regulates cell motility, survival, proliferation, angiogenesis and cell invasion [21, 22]. The antineoplastic potential of snake venom disintegrins have been shown in several studies, e.g. contortrostatin (disintegrin isolated from *Agkistrodon contortrix* venom) [23], saxatilin (disintegrin from *Gloydius saxatilis* venom) [24], PAIEM (disintegrin isolated from *Echis multisquamatis* venom) [25] and a disintegrin from *Crotalus durissus collilineatus* venom [26]. The disintegrins act by inhibiting angiogenesis, invasion and migration of cancer cells. The finding of disintegrin in the Malaysian *T. purpureomaculatus* venom is significant as the peptide could be a promising local source of anticancer candidate that has not been characterized previously. 

In this study, the Malaysian *T. purpureomaculatus* venom was suggested to be cytotoxic to a wider range of cancer cell lines including those of the human breast, lung, colon and prostate. The selective anticancer activity, if any, could be partly contributed by the disintegrin present in the venom. Hence, this study aimed to investigate the cytotoxicity of Malaysian *T. purpureomaculatus* venom across four common human cancerous and the corresponding normal cell lines. In addition, the disintegrin was purified from the venom, and its amino acid sequence as well as anticancer properties were characterized. 

## Methods

### Venom Sample

The venom of mangrove pit viper (*Trimeresurus purpureomaculatus*, MTP) was a pooled sample from ten adult snake specimens (four males; six females) from Peninsular Malaysia. The venom obtained was freeze-dried and stored at −20 °C until use. Venom stock used in each cytotoxic experiment was prepared freshly from lyophilized venom and subjected to a quick spin for 15 seconds prior to the treatment. 

### Chemicals and Materials

All chemicals and reagents used in the studies were of analytical grade. 3,(4,5-dimethythiazol-2-yl)-2,3-diphenyl tetrazolium bromide (MTT) was purchased from Sigma Aldrich (Missouri, USA) and dimethyl sulfoxide (DMSO) was supplied by Merck (Darmstadt, Germany). Trypsin-EDTA was supplied by Nacalai Tesque (Kyoto, Japan). The positive control in the MTT assay was 5-fluorouracil (5-FU) (Sigma-Aldrich, Missouri, USA). The reversed-phase HPLC column LiChrospher® WP 300 RP-18 (5 μm) was purchased from Merck (Darmstadt, Germany) and HPLC solvents were from (Thermo Scientific, Massachusetts, USA). Trifluoroacetic acid (TFA) was purchased from Sigma Aldrich (Missouri, USA). Ammonium bicarbonate, dithiothreitol (DTT) and iodoacetamide (IAA), which were used in protein digestion, were purchased from Sigma-Aldrich (Missouri, USA), Trypsin protease (MS grade) was purchased from Thermo Scientific Pierce^™^ (Massachusetts, USA) and desalting C_18_ pipette tips was purchased from Merck Millipore ZipTip^®^ (MilliporeSigma, Massachusetts, USA). Protein ladder of PM2700 ExcelBand™ 3-color Broad Range Protein Marker was purchased from SMOBIO Technology, Inc. (Hsinchu, Taiwan).

### Cell Culture

Both normal and cancer cell lines were obtained from American Type Culture Collection (ATCC, Virginia, USA). MCF7 (ATCC^®^ HTB-22^™^; human breast adenocarcinoma cell line), HT-29 (ATCC^®^ HTB-38^™^; human colon colorectal adenocarcinoma cell line), A549 (ATCC^®^ CCL-185^™^; human lung carcinoma cell line) and PC-3 (ATCC^®^ CRL-1435^™^; human prostate adenocarcinoma cell line) were used as the cancer cell panel, while 184B5 (ATCC^®^ CRL-8799^™^; human breast normal cell line), CCD-18Co (ATCC^®^ CRL-1459^™^; human colon normal cell line), MRC5 (ATCC^®^ CCL-171^™^; human lung normal cell line) and RWPE-1 (ATCC^®^ CRL-11609^™^; human prostate normal cell line) were used as the normal cell panel in the cytotoxic assay. 

MCF7 and HT-29 cell lines were cultured in Dulbecco’s modified Eagle’s medium (DMEM) (Nacalai Tesque, Kyoto, Japan), supplemented with 10% fetal bovine serum (FBS) (TICO Europe, Amstelveen, Netherlands) and 100 µg/mL penicillin-streptomycin (Nacalai Tesque, Kyoto, Japan), while A549 and PC-3 were maintained using RPMI 1640 medium with L-glutamine (Lonza, Verviers, Belgium), with 10% FBS and 100 µg/mL penicillin-streptomycin. Normal cell lines such as CCD-18Co and MRC5 were maintained in Eagle’s minimum essential medium (EMEM) (Sigma-Aldrich, Missouri, USA), with 10% FBS and 100 µg/mL penicillin-streptomycin. The 184B5 was maintained in mammary epithelial cell growth medium (MEGM) with bullet kit (Lonza, Basel, Switzerland), supplemented with 10% FBS and 100 µg/mL penicillin-streptomycin, while RWPE-1 was maintained in Keratinocyte-SFM (serum-free medium) (Thermo and Scientific, Massachusetts, USA), with only 100 µg/mL penicillin-streptomycin added to the medium. The cell lines were cultured in a 5% carbon dioxide (CO_2_) incubator (Shel Lab, Oregon, USA) at 37 ºC, with pH of 7.2 - 7.5 and relative humidity of about 95%. The growth of cells was monitored routinely by observing cell morphology under an inverted microscope (Leica Microsystems, Wetzlar, Germany). All of the cell-related work was carried out using aseptic techniques under sterile condition. 

### Cell Viability Assay

Cell viability was studied with the 3-(4,5-dimethylthiazol-2-yl)-2,5-diphenyltetrazolium bromide (MTT) assay. The cells of the respective cell lines were detached from the culture flasks using an appropriate amount of trypsin-EDTA and desired number of cells were seeded in a 96-well microplate (15,000-150,000 cells/mL) for overnight incubation. The attached cells were then treated with serial dilutions (ranging from 0.10-31.62 μg/mL) of *T. purpureomaculatus* venom for 72 hours. After treatment, 10% of MTT reagent was added into each well without removing the previous medium. The plate was covered with aluminum foil and returned to the CO_2_ incubator for 3 hours incubation. The MTT solution was pipetted out gently after the incubation and 200 μL of dimethyl sulfoxide (DMSO) was added into each well to solubilize the formazan crystal. Absorbance was measured using Hidex Plate CHAMELEON™V (Hidex, Turku, Finland) multilabel microplate reader at 570 nm. The median inhibition concentration (IC_50_) was determined from the dose-response curve plotted with percentage of cell viability against venom concentration (μg/mL). In the assay, 5-fluorouracil (5-FU) was used as a positive reference. The percentage of cell viability was calculated with the following formula:

$$Cell\;viability\;(\%)=\frac{Average\;OD\;of\;treated\;cells-Average\;OD\;of\;blank}{Average\;OD\;of\;control\;cells-Average\;OD\;of\;blank}\times 100\%$$

### Selectivity Index Determination 

The degrees of selective cytotoxicity of the venom in cancer cell lines were indicated with selectivity index (SI), determined as follows:

$$Selectivity\;index\;(SI)=\frac{\left({IC}_{50}\;in\;normal\;cell\;line\right)}{\left({IC}_{50}\;in\;cancer\;cell\;line\right)}$$

### **Fractionation of *T. purpureomaculatus* Venom and Bioassay-Guided Cytotoxicity Study**

Two hundred microliters of *T. purpureomaculatus* venom solution (10 mg/mL in ultrapure water) were injected into LiChrosper® WP 300-RP-18 reversed-phase column (5 μm column particle size) through the Shimadzu LC-20AD high performance liquid chromatography system. The column was pre-equilibrated with solvent B [0.1% trifluoroacetic acid (TFA) in acetonitrile] followed by solvent A (0.1% TFA in water). The elution began with the stepwise linear gradient (0-5% of B for 10 min, followed by 5-15% B for 20 min, 15-60% B for the next 180 minutes, 60-70% B for 10 minutes and 75-100% of B over 245 min) in 0.1% TFA in acetonitrile (ACN) for 245 minutes (flow rate: 1 mL/min). Protein elution was monitored at 215 nm. 

The protein fractions were collected manually, subsequently freeze-dried and stored at −20 °C until use. Each protein fraction was reconstituted in ultrapure water and the protein concentration was estimated by Nanodrop Spectrophotometer 2000 (ThermoFisher™, Massachusetts, USA) prior to the experiment. The cytotoxic fraction of *T. purpureomaculatus* venom was screened with a bioassay-guided method modified from Shahbazi [27]. The cytotoxic activities of the venom fraction were tested at a standard dose of 20 µg each on human breast cancer cell line (MCF7) according to the cell viability assay described above. The amount of protein from each venom fraction tested (20 µg) was referred from a previous study by Bradshaw [28]. Cell images were captured 72 hours after treatment using inverted microscope (Leica Microsystems, Wetzlar, Germany).

### Sodium Dodecyl Sulphate-Polyacrylamide Gel Electrophoresis (SDS-PAGE)

Gel electrophoresis was carried out according to the protocol of Laemmli [29]. Approximately 10 μg of protein fraction was loaded onto a 15% acrylamide gel and the electrophoresis was carried out under reducing conditions at 90 V for 2 hours. Protein ladder PM2700 ExcelBand™ 3-color Broad Range Protein Marker was used as molecular weight standards in the electrophoresis (5-245 kDa). Coomassie Brilliant Blue R-250 staining was used for the visualization of protein. 

### Protein Digestion and Mass Spectrometry Analysis of the Cytotoxic-Contributing Fraction of MTP Venom

The most cytotoxic protein fraction of MTP venom was subjected to in-solution tryptic digestion and nano-ESI LC-MS/MS analysis as previously reported [30]. Ten micrograms of the protein (10 µL of 1 mg/mL protein concentration) first underwent reduction and alkylation by addition of 15 μL of 50 mM ammonium bicarbonate and 1.5 μL of 100 mM dithiothreitol (DTT). The mixture was heated at 95 ºC for 5 min before addition of 3 μL of 100 mM iodoacetamide (IAA). The mixture was later incubated in dark at room temperature for another 20 min. Digestion was carried out by adding one microliter of 0.1 mg/mL trypsin solution to the reaction tube and incubated at 37 ºC for 3 hours. Additional 1 µL of trypsin solution was added for overnight incubation at 30 ºC. After digestion, the digested peptides were desalted for removal of salts and contaminants. 

The desalted peptides (approximately 10 µg) were then reconstituted in 7 µL of 0.1% formic acid in water. One microliter of the solution (containing approximately 1.4 µg of peptides) was subjected to nano-electrospray ionization MS/MS via Agilent 1260 HPLC-Chip/MS Interface coupled with Agilent 6550 Accurate-Mass Q-TOF LC/MS system (Agilent Technologies, Santa Clara, California, USA). The sample peptides were separated in a large capacity chip Zorbax 300 Å, C18, 160 nl enrichment column, 75 μm × 150 mm analytical column and 5 μm particles (Agilent part no. G4240-62010). Parameters were set as follows: injection volume at 1 µL per sample, flow rate from capillary pump at 4 µL/min and 0.4 µL/min from Nano pump (G2226A), gradient used: 5-50% solution B (0.1% formic acid in acetonitrile) for 11 min, 50-70% B for next 4 min, and 70% B for 3 min and ion polarity was set to positive ionization mode. Drying gas flow and temperature were set at 11 L/min and 290 ºC. Fragmentor voltage was set at 175 V while for capillary voltage, it was set at 1800 V. MS scan range of 200-3000 *m/z* and MS/MS scan range of 50-3200 *m/z* were acquired in the tandem mass spectrometry mode, with precursor charge selection set as doubly charge state and above with the exclusion of precursor 1221.9906 m/z (z = 1) for internal mass calibration and reference ions set at 299.2944 (z = 1). Data with a MH^+^ mass range between 50 and 3200 were extracted and analyzed in Agilent Spectrum Mill MS Proteomics Workbench software packages (Agilent Technologies, Santa Clara, CA, USA). Setting for cysteine carbamidomethylation is set as a fixed modification, while methionine oxidation is set as variable modification. 

The mass spectrometry derived peptide masses were searched against a non-redundant NCBI database of Serpentes (taxid: 8570) combined with the venom-gland transcriptome database of *T. purpureomaculatus*. The protein identification was validated by the following filters: protein score > 20, peptide score > 10 and score peak intensity (SPI) > 70%. Proteins with “Distinct Peptide” ≥ 2 were considered significant matches. 

### Bioinformatic Analyses 

**Sequence analysis of disintegrin**

Multiple sequence alignment of disintegrins was performed using MUSCLE program and Jalview software v2.11 to indicate the region of similarity. The percentage of similarity was calculated using EMBL-EBI Clustal Omega (https://www.ebi.ac.uk/Tools/msa/clustalo/). The disintegrin sequence of Malaysian *Trimeresurus purepureomaculatus* (MTP) venom was derived from the venom-gland transcriptome (entry is available in NCBI GenBank database, accession ID: QJA41976.1). Other related disintegrin sequences were retrieved from Uniprot Knowledgebase (https://www.uniprot.org/) [31]. 

**In silico physicochemical characterization of disintegrin**

The physicochemical properties of the disintegrin from MTP venom were characterized using the Expasy ProtParam online tool (https://web.expasy.org/protparam/) according to Roly et al. [32]. Parameters including theoretical molecular weight, isoelectric point (pI), instability index and grand average of hydropathicity (GRAVY) value were determined.

### Statistical Analysis

The median inhibition concentration (IC_50_) value was determined using GraphPad Prism 5 statistical software (GraphPad Software Inc., California, USA) and the values were expressed as mean ± S.E.M. of three replicates. Comparative data were statistically analyzed using Student’s unpaired t-test (at 95% confidence interval) with GraphPad Prism 5 software (GraphPad Software Inc., California, USA). 

## Results

### **Cytotoxicity of *Trimeresurus purpureomaculatus* Venom**

**Figure 1.**
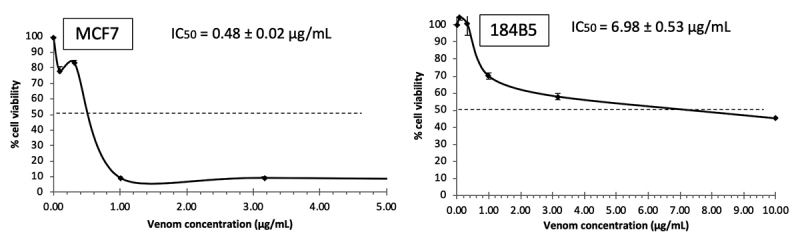
Dose-dependent growth inhibitory effect of *Trimeresurus purpureomaculatus* (MTP) venom in human breast cell lines. Median inhibition concentrations (IC_50_) were determined from the dose-response curve. Values were presented as means ± S.E.M. (*n* = 3). MCF7: human breast cancer cell line, 184B5: human breast normal cell line.

MTP venom exhibited dose-dependent cytotoxic effects toward all cancer cell lines tested (median inhibition concentrations, IC_50_ = 0.42−2.50 µg/mL). The effects were generally stronger in the cancer cells compared to the corresponding normal cells (IC_50_ = 0.70-6.98 µg/mL) (Table 1). The venom cytotoxicity was most potent to the colorectal adenocarcinoma cell line (IC_50_ = 0.42 ± 0.06 µg/mL), followed by the breast cancer cell line (IC_50_ = 0.48 ± 0.02 µg/mL) and prostate cancer cell line (IC_50_ = 1.60 ± 0.18 µg/mL). The venom was least cytotoxic to the lung cancer cell line (IC_50_ = 2.50 ± 0.20 µg/mL). The venom was also significantly more potent compared to 5-FU in inhibiting the growth of breast, lung and prostate cancer cells (*p* < 0.05). In comparison to the IC_50_ in normal cell lines, the cytotoxic effect was not selective toward colon, lung and prostate cancer cells as indicated by a selectivity index (SI) ≤ 10. The selectivity index of the venom was, however, much higher (SI = 14.54) for the breast cancer cell line (MCF7), implying that the venom was 15-fold more cytotoxic to MCF7 than it was to the corresponding normal cell line (184B5) (Table 1, Figure 1). 

**Table 1.** Median inhibition concentrations (IC_50_) (µg/mL) and selectivity indices (SI) of Malaysian *T. purpureomaculatus* (MTP) venom for different cell lines after 72-hour treatment in comparison to 5-fluorouracil (5-FU).

	MTP		5-FU	
Cell Lines	**IC_50_ (µg/mL)**	**SI**	**IC_50_ (µg/mL)**	**SI**
**Breast**				
MCF7	0.48 ± 0.02	14.54	8.26 ± 0.77	0.16
184B5	6.98 ± 0.53		1.34 ± 0.14	
**Colon**				
HT-29	0.42 ± 0.06	1.67	0.12 ± 0.02	>258.33
CCD-18Co	0.70 ± 0.15		> 31.62	
**Lung**				
A549	2.50 ± 0.20	1.44	> 31.62	NA
MRC5	3.61 ± 0.14		> 31.62	
**Prostate**				
PC3	1.60 ± 0.18	1.19	12.83 ± 1.52	0.22
RWPE-1	1.91 ± 0.53		2.79 ± 0.66	

IC_50_ values were derived from triplicates ± S.E.M.

Selectivity index ≥ 10 is considered as the threshold for a compound having cancer-selective cytotoxic effect [28].

NA: not available, MCF7: human breast cancer cell line, 184B5: human breast normal cell line, HT-29: human colon cancer cell line, CCD-18Co: human colon normal cell line, A549: human lung cancer cell line, MRC5: human lung normal cell line, PC3: human prostate cancer cell line, RWPE-1: human prostate normal cell line.

### **Cytotoxicity of the Chromatographic Fractions of *Trimeresurus purpureomaculatus* Venom**

**Figure 2.**
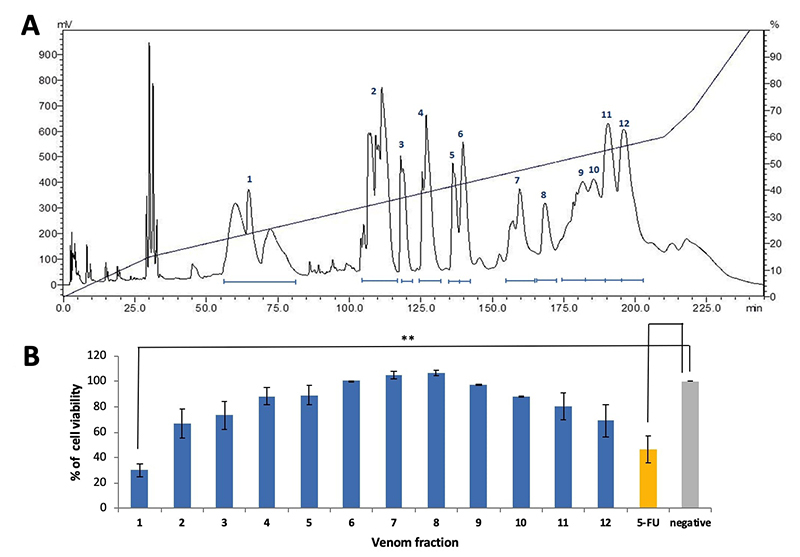
**(A)** C_18_ reversed-phase high performance liquid chromatography of venom from the Malaysian *T. purpureomaculatus*. **(B)** Cell viability of human breast cancer cells (MCF7) after 72-hour treatment with the HPLC fractions of Malaysian *T. purpureomaculatus* venom. Positive and negative controls were 5-fluorouracil (5-FU) and treatment-free, respectively, in the assay.

MTP venom was resolved using C_18_ reversed-phase high performance liquid chromatography (RP-HPLC) into 12 fractions as shown in Figure 2A. The cytotoxic effect of each fraction was further tested on MCF7 cell line in view of the high selectivity index (SI) of MTP venom in breast cancer cell line. MCF7 cells treated with protein from Fraction 1 (20 µg) showed the most potent inhibitory effect (cell viability reduced by 65%) compared to the non-treated control (**p < 0.01). Proteins from Fraction 2 and 12 exhibited moderate cytotoxic effect (cell viability reduced by 25−30%) whereas other fractions did not show significant cytotoxicity in the MCF7 cell line (Figure 2B). In comparison, 5-FU at IC_50_ of 9 µg/mL induced a 50% reduction in the cell viability study. 

### **Identification and Cytotoxicity of Disintegrin from *Trimeresurus purpureomaculatus* Venom**

**Figure 3.**
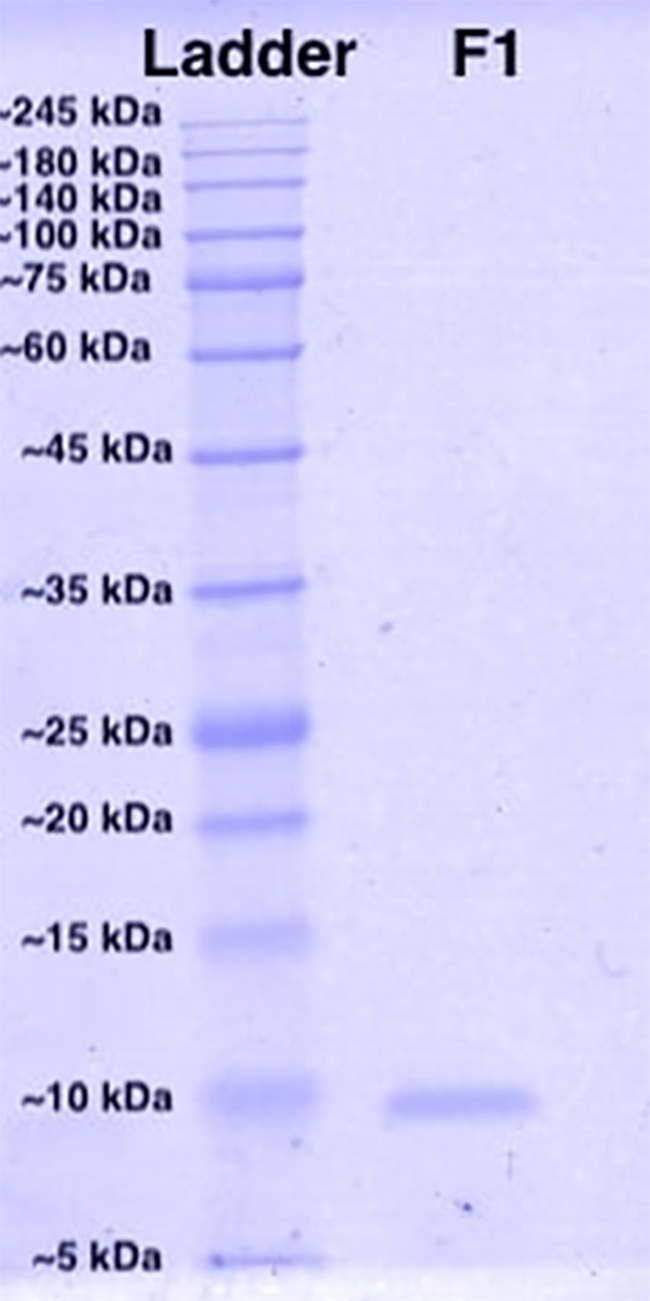
Protein content of Fraction 1 (F1) of Malaysian *T. purpureomaculatus* venom was validated under 15% reducing gel electrophoresis. Protein ladder PM2700 ExcelBand™ 3-color Broad Range Protein Marker was used for molecular weight standards (5−245 kDa).

Figure 3 shows the SDS-PAGE profile of the cytotoxic protein isolated in Fraction 1. A homogenous protein band was observed and the molecular mass was estimated to be 7585 Da by its relative migration distance (Rf). LC-MS/MS identified Fraction 1 to contain only one protein, which is a disintegrin of snake venom and it is termed as “purpureomaculin” in the present study. The mass spectrometry data file of purpureomaculin is available in Additional file 1, and the primary data was deposited to the ProteomeXchange Consortium via the iProX partner repository [33] with the dataset identifier PXD018463. 

**Figure 4.**
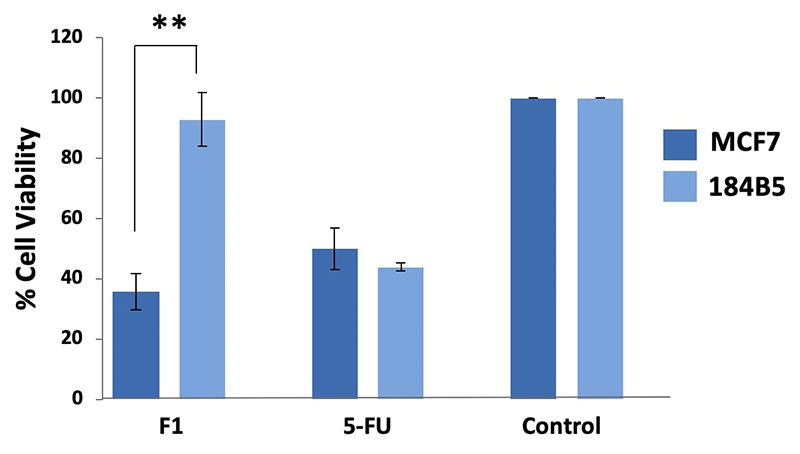
Cell viability of human breast cell lines, MCF7 (cancerous) and 184B5 (normal) after 72-hour treatment with 20 µg of Fraction 1 (F1, purpureomaculin) purified from *Trimeresurus purpureomaculatus* venom. Positive control: 5-fluorouracil (5-FU); negative control: treatment-free well.

**Figure 5.**
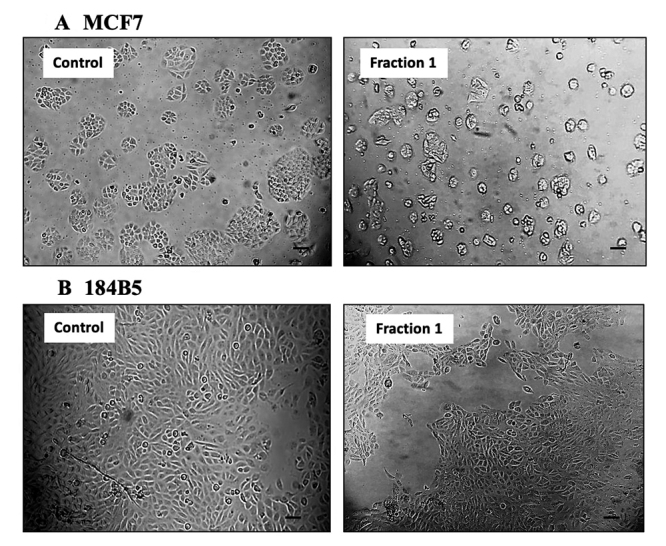
Morphological changes in human breast cell lines, **(A)** MCF7 (cancerous) and **(B)** 184B5 (normal) after 72-hour treatment of Fraction 1 (purpureomaculin) of *Trimeresurus purpureomaculatus* venom. Scale bars = 30 µm.

Purpureomaculin at 20 µg significantly inhibited the growth of MCF7 cell line by 65% (**p < 0.01), while no significant cytotoxic effect was observed in the corresponding normal breast cells (184B5 cell line) (Figure 4). Microscopic examination revealed the presence of detached, clumped and rounded floater cells in the purpureomaculin-treated breast cancer cells following a treatment period of 72 hours. On the contrary, the normal cell line (184B5) treated with purpureomaculin was observed to remain healthy and viable, with slight reduced growth compared to the untreated control (Figure 5). 

### **Sequence Analysis and *in silico* Characterization of Purpureomaculin, the Cytotoxic Disintegrin from *T. purpureomaculatus* Venom**

The tryptic peptides of purpureomaculin (K)LLPGAQCGEGLCCDQCSFMKK(G) and (R)ARGDDLDDYCNGISAGCPR(N) from Fraction 1 were matched to the disintegrin transcript “CL53.Contig5_CP” from the Malaysian *T. purpureomaculatus* venom-gland transcriptomic database. A disintegrin with full amino acid sequence was identified. It is a mature chain polypeptide of 73 amino acid residues: EAGEDCDCGSPANPCCNAATCKLLPGAQCGEGLCCDQCSFMKKGTICRRARGDDLDDYCNGISAGCPRNPLHA. 

**Figure 6.**
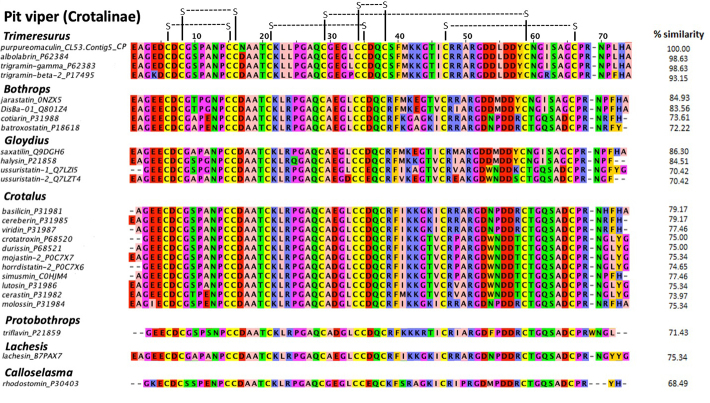
Multiple sequence alignment of purpureomaculin (CL53.Contig5_CP) with other disintegrin sequences of Crotalinae. Purpureomaculin is a disintegrin isolated from the Malaysian *Trimeresurus purpureomaculatus* venom, while other disintegrin sequences were obtained from UniProtKB. The percentages of similarity (% similarity) of disintegrin sequences in comparison to purpureomaculin were calculated using Omega Clustal software. Zappo color scheme indicates residue properties: pink: aliphatic/hydrophobic; orange: aromatic; blue: positive; red: negative; green: hydrophilic; magenta: proline/glycine (conformationally special); yellow: cysteine. Disulfide bonds were illustrated in black connecting lines.

Multiple sequence alignment showed that purpureomaculin is highly homologous (98.6%) with albolabrin from *Trimeresurus albolabris* venom and trigramin-gamma from *Trimeresurus gramineus* venom, where purpureomaculin varies from albolabrin and trigramin-gamma by only one amino acid at the 17^th^ position of the sequence (substitution of Asp to Asn in purpureomaculin) (Figure 6). Six disulfide bonds could be formed within the sequence, pairing cysteine residues at the following positions: (6, 15), (8, 16), (21, 35), (29, 59), (34, 38), (47, 66). Purpureomaculin also harbored the tripeptide RGD, a short sequence motif of biological interest formed by amino acid residues 51−53. Compared with the disintegrin sequences of other viperids, high similarity (> 70%) was observed in phylogenetically related pit viper genera and species, e.g. *Trimeresurus* complex. 

Table 2 shows the physicochemical properties of purpureomaculin analyzed *in silico* using the ProtParam Tool. Purpureomaculin has an estimated molecular weight of 7572.52 Da and is weakly acidic (pI = 4.79). 

**Table 2.** Physicochemical properties of purpureomaculin from the Malaysian *Trimeresurus purpureomaculatus* venom.

Properties	Values
Residues	73
Mr	7572.52
pI	4.79
Instability Index (II)^a^	25.23
Stability	Stable
Aliphatic Index (AI)^b^	48.36
GRAVY^c^	-0.390
Extinction coefficients*^d^	2240
Extinction coefficients* (reduced)^e^	1490

Mr: Molecular weight (Da); pI: Isoelectric point; GRAVY: Grand average of hydropathicity.

^a^ Estimate of protein stability (instability index of <40 is predicted as stable).

^b^ Relative volume occupied by aliphatic side chains (alanine, valine, isoleucine, and leucine). Positive factor denotes for the increase of thermostability.

^c^ Sum of hydropathy values of all amino acid, divided by total number of residues in sequence.

*Amount of light a protein absorbs at a certain wavelength. Extinction coefficient unit is in M^-1^cm^-1^, measured at 280 nm in water.

^d^ Assuming all pairs of cysteine residues appears as half cystines.

^e^ Assuming all cysteine residues are reduced/no cysteine appears as half cystine.

## Discussion

In this study, the potent cytotoxicity of Malaysian *T. purpureomaculatus* venom was shown in a panel of cancer (MCF7, HT-29, PC3, A549) and normal (184B5, CCD-18Co, RWPE-1, MRC5) cell lines. MTP venom was particularly toxic to breast cancer cell line (MCF7) and colon cell lines (HT-29 and CCD-18Co), indicating high specificity of the venom toward these tested cells. The venom also demonstrated selective cytotoxicity, particularly in the human breast cancer cells (MCF7). This suggests that the venom contains toxin(s) or protein(s) that could be cancer-specific in breast tumor, thus potentiating the venom cytotoxicity at a lower dose in the cancer cells compared to the corresponding normal cells. The cytotoxic effect of Malaysian *T. purpureomaculatus* venom also appeared to be more potent than other pit viper venoms, including venoms of *Protobothrops flavoviridis* [35], *Trimeresurus macrops* and *Trimeresurus hageni* [36], albeit different human cell lines were utilized in each study.

Further investigation revealed that most of the venom components (fractionated with HPLC) showed variable anticancer activity, implying that the overall venom cytotoxicity could be a result of synergistic interactions of multiple components in the venom. The most potent anti-neoplastic activity was found in the protein fraction containing snake venom disintegrin. This was a purified protein validated through SDS-PAGE and LC-MS/MS, and termed purpureomaculin in line with the naming of most snake venom-derived disintegrin protein (prefix “species-” + post-fix “-in”). The finding of purpureomaculin anticancer activity is consistent with earlier studies that reported the inhibitory activity of snake venom disintegrins on the proliferation, metastasis and adhesion of cancer cells [21, 25, 37]. The selective cytotoxic effect of purpureomaculin in breast cancer cells (MCF7) corroborates the selective cytotoxicity of MTP venom in the same cancer cell line observed in this study. 

The present findings varied when compared with previous studies on other cancer cells using *T. purpureomaculatus* venoms from Malaysia and Thailand [17, 18]. IC_50_ values with wide discrepancy were observed between previous studies (Malaysian *T. purpureomaculatus* IC_50 (72 hours)_: 15.99−29.43 µg/mL in human colon cell lines [18]; Thai *T. purpureomaculatus* (IC_50 (48 hours)_: 0.25−5.24 µg/mL in human neuroblastoma, cervical, colon, breast, bladder, glioblastoma and kidney cell lines [17]) and the present work (Malaysian *T. purpureomaculatus*: IC_50 (72 hours)_: 0.42-6.98 µg/mL in breast, lung, colon and prostate cell lines) (Table 3). Nevertheless, the cytotoxic activity of the present Malaysian *T. purpureomaculatus* venom in human colon cells appeared to be comparable to the Thai venom sample reported by Ozverel [17]. In contrast, much higher IC_50_ values (by a difference of > 10 folds) in human colon cells were reported previously in the other Malaysian *T. purpureomaculatus* venom sample [18]. The discrepancy could be due to the variation in venom composition, the use of different colon cell lines, or the influence of the condition of venom preparation. In our experience, repeated freeze-thaw cycles of the MTP venom led to inconsistent and somewhat deteriorating venom cytotoxic activity (unpublished); hence, all venom samples used in the present study were freshly reconstituted from lyophilized stock sample prior to treating the cells in the MTT experiment. The additional measure, presumably, avoided any possible protein degradation [38] or inactivation of the venom bioactive components by unfavorable pH or temperature [39]. 

In addition, previous studies had demonstrated variable anticancer activities for venom disintegrin from pit vipers. For instance, lebein (disintegrin isolated from *Macrovipera lebetina* venom) has been shown to significantly reduce the cell viability of colon adenocarcinoma cell lines after 72-hour incubation [40]. A more recent study demonstrated that disintegrins isolated from *Cerastes cerastes* venom displayed varying IC_50_ values (1.60−8.17 µg/mL) in SHSY5Y neuroblastoma cell line [17]. Tzabcanin (disintegrin isolated from *Crotalus simus tzabcan* venom) exhibited low cytotoxicity against Colo-205 cells and was not cytotoxic to MCF7 cells [41]. On the contrary, the disintegrin purpureomaculin of *T. purpureomaculatus* was found to be potent in inhibiting the growth of MCF7 cells (current study). The anticancer activities of disintegrin from many closely related species such as trigramin (disintegrin isolated from *Trimeresurus gramineus* venom) and albolabrin (disintegrin isolated from *Trimeresurus albolabris* venom) remain unknown to date, and warrant investigation for insights in the anticancer potential of the venom peptide from this large Asiatic pit viper complex. 

**Table 3.** Comparison of cytotoxicity studies of *T. purpureomaculatus* venoms from different locations (Malaysia and Thailand).

Species	*Trimeresurus* *purpureomaculatus* (current work)			*Trimeresurus* *purpureomaculatus* [18]			*Trimeresurus* *purpureomaculatus* [34]		
Origin	Malaysia (wild)			Malaysia			Thailand (kept in Turkey)		
Whole venom study									
Treatment duration	72 hours			72 hours			48 hours		
Tissue-organ and cell line	Cancerous	Normal	Selectivity	Cancerous	Normal	Selectivity	Cancerous	Normal	Selectivity
Breast	MCF7, IC_50_ = 0.48 ± 0.02 µg/mL	184B5, IC_50_ = 6.98 ± 0.53 µg/mL	Yes (S.I. = 14.54)	N.D.	N.D.	N.D.	MCF7, IC_50 s_> 50 µg/mL MDA-MB-231, IC_50_ = 1.77 ± 0.21 µg/mL	N.D.	N.D.
Colon	HT-29, IC_50_ = 0.42 ± 0.06 µg/mL	CCD-18Co, IC_50_ = 0.70 ± 0.15 µg/mL	No	SW480, IC_50_ = 29.43 ± 0.48 µg/mL SW620, IC_50_ = 23.19 ± 1.57 µg/mL	CCD-18Co, IC_50_ = 15.99 ± 1.20 µg/mL	No	CaCo-2, IC_50_ = 3.10 ± 1.40 µg/mL	N.D.	N.D.
Lung	A549, IC_50_ = 2.50 ± 0.20 µg/mL	MRC5, IC_50_ = 3.61 ± 0.14 µg/mL	No	N.D.	N.D.	N.D.	A549, IC_50_ = 4.65 ± 0.19 µg/mL	N.D.	N.D.
Prostate	PC3, IC_50_ = 1.60 ± 0.18 µg/mL	RWPE-1, IC_50_ = 1.91 ± 0.53 µg/mL	No	N.D.	N.D.	N.D.	N.D.	N.D.	N.D.
Nerve	N.D.	N.D.	N.D.	N.D.	N.D.	N.D.	SHSY5Y (neuroblastoma), IC_50_ = 0.25 ± 0.05 µg/mL U87MG (glioblastoma),IC_50_ = 1.32 ± 0.03 µg/mL	N.D.	N.D.
Cervix	N.D.	N.D.	N.D.	N.D.	N.D.	N.D.	HeLa, IC_50_ = 2.15 ± 1.50 µg/mL	N.D.	N.D.
Urinary bladder	N.D.	N.D.	N.D.	N.D.	N.D.	N.D.	253-JBV, IC_50_ = 5.24 ± 0.77 µg/mL	N.D.	N.D.
Kidney	N.D.	N.D.	N.D.	N.D.	N.D.	N.D.	N.D.	HEK293, IC_50_ = 2.04 ± 0.21 µg/mL	N.D.
Venom fraction study									
Fractionation method	Reversed-phase HPLC			Size-exclusion & reversed-phase HPLC			Reversed-phase HPLC		
Treatment duration	72 hours			72 hours			48 hours		
Tissue-organ and cell line	Cancerous	Normal	Selectivity	Cancerous	Normal	Selectivity	Cancerous	Normal	Selectivity
Breast	MCF7; disintegrin at 20 µg Reduced cell viability by 65%	184B5; disintegrin at 20 µg Did not significantly reduced cell viability	Yes	N.D.	N.D.	N.D.	N.D.	N.D.	N.D.
Colon	N.D.	N.D.	N.D.	SW480; LAAO IC_50_ = 13.56 ± 0.58 µg/mL SW620; LAAO IC_50_ = 13.17 ± 0.77 µg/mL	CCD-18Co; LAAO IC_50_ = 14.98 ± 2.67 µg/mL	No	N.D.	N.D.	N.D.
Nerve	N.D.	N.D.	N.D.	N.D.	N.D.	N.D.	SHSY5Y (neuroblastoma); BPP-RP IC_50_ = 37.01 ± 3.30 µg/mL & 78.39 ± 5.38 µg/mL PLA_2_ IC_50_ = 43.79 ± 3.40 µg/mL peptide-rich fraction IC_50_ = 34.07 ± 0.07 µg/mL	N.D.	N.D.

HPLC: high performance liquid chromatography, IC_50_: median inhibition concentration, S.I.: selectivity index, N.D.: not determined, LAAO: L-amino acid oxidase, BPP-RP: bradykinin-potentiating peptide, PLA_2_: phospholipase A_2_.

The multiple sequence alignment revealed that the venom disintegrins of pit vipers (Crotalinae) are mainly of intermediate length with 12 conserved cysteine residues, forming 6 disulfide bonds that support the folding and stability of the protein [42]. Disintegrins from *Trimeresurus*, *Bothrops*, *Gloydius*, *Crotalus*, *Protobothrops* and *Lachesis* complexes in particular showed a higher rate of amino acid conservation, indicating the phylogenetic relationship among different genera within the Crotalinae subfamily. At the intra-generic level of *Trimeresurus*, purpureomaculin sequence was found almost identical (similarity: 98.6%) to albolabrin and trigramin-gamma (disintegrins from *Trimeresurus albolabris* and *Trimeresurus gramineus*). Like albolabrin and trigramin, purpureomaculin is structurally categorized as the medium-sized disintegrins which contain about 70 amino acids and 6 disulfide cysteine bonds, as described by Calvete [42]. The high similarity of purpureomaculin to these disintegrins indicates structural and functional resemblance between these disintegrins, and that purpureomaculin is likely to exert its cytotoxic activity similar to trigramin and albolabrin by inhibiting cell-matrix adhesion through the binding of RGD tripeptide domain to the cellular integrin receptors [43, 44]. One remarkable difference of purpureomaculin is the amino acid substitution at the 17^th^ position. It is noted that amino acid at this position is highly conserved throughout the pit viper genera, and the substitution of the aspartic acid (D) to the neutral asparagine (N) in purpureomaculin may have a unique evolutionary implication on the role of the protein expressed. The consequence of the amino acid substitution deserves further investigation. 

The sequence similarities between purpureomaculin and other disintegrin sequences are lower (69−86%) when comparing with venom disintegrins of different genera other than *Trimeresurus*. All pit viper disintegrin sequences (including purpureomaculin) contains RGD-flanking motif, a tripeptide domain known to bind to a wide range of integrin receptors. In particular, disintegrins with RGD tripeptides were known to bind key integrin receptors such as α2β1 [45], α4β1 [46], αvβ3 [47, 48] and αvβ5 [49], leading to inhibition of integrin-mediated functions in cancer cells. Among these, the high expression of integrins αvβ3 and αvβ5 are commonly associated with tumorigenesis [50]. A distinct feature of MCF7 cell line is that it lacks integrin αvβ3 receptor, but showed hyperexpression of integrins αvβ5 receptor [51]. We hypothesized that the selective cytotoxicity of purpureomaculin is at least in part mediated through the binding of integrin receptors (e.g. αvβ5 receptor) that leads to the inhibition of cancer cell adhesion, angiogenesis and metastasis. This explains the microscopic observation in the present study, where purpureomaculin caused significant detachment of the treated breast cancer cells (MCF7), forming round-shape floaters while sparing the architecture of the normal breast cells (184B5). 

In addition, the primary sequence of purpureomaculin was analyzed *in silico* to further understand the physicochemical properties of the identified disintegrin. Purpureomaculin was computed to have a molecular weight of 7572.5 Da, similar to that determined from the reducing SDS-PAGE of the protein (7585 Da). The molecular mass determined, however, may be slightly varied from that examined by intact mass profiling using a top-down mass spectrometry analysis. The intact mass profiling of purpureomaculin hence should be carried out in the future to establish the exact mass. On the other hand, the computed instability index (II) that falls below 40 indicates that purpureomaculin is theoretically stable *in vitro*. The instability index could be applied to estimate the *in vivo* half-life of a protein [52]: Proteins whose II < 40 have an *in vivo* half-life beyond 16 h, whereas proteins whose II > 40 are usually eliminated faster from the body with an *in vivo* half-life of less than 5 h [53]. The II value obtained for purpureomaculin in this work was about 25, predicting a long *in vivo* half-life beyond 16 h for this disintegrin. The aliphatic index (AI) represents the relative volume of aliphatic side chains (alanine, valine, isoleucine and leucine) in the amino acid sequence of a protein. The AI value computed for purpureomaculin reflects high protein thermostability. On the other hand, the GRAVY parameter predicts for the feature hydrophobic (positive values) or hydrophilic (negative values) of a protein. Purpureomaculin has a negative value of GRAVY, implying that it is more of a hydrophilic protein - this is also consistent with the early elution of this protein from the reversed-phase HPLC. Together, the physicochemical parameters predict that purpureomaculin is a relatively stable protein with an extended *in vivo* half-life, and readily dissolves in a polar solvent. Moreover, the small molecular size of the disintegrin peptide (7572.52 Da) implies that it is likely less antigenic, more accessible to cancer cell environment and amenable to structural modification e.g. nano-carrier conjugation in the advanced development of peptide-based anticancer agent [54, 55].

## Conclusion

The present study demonstrated the potent cytotoxicity of the Malaysian *T. purpureomaculatus* venom and unveiled its selective anticancer activity in the human breast cancer cell line (MCF7). Purpureomaculin, a disintegrin identified from the Malaysian *T. purpureomaculatus* venom, was found to be most cytotoxic and selective among several protein fractions of the venom, presumably due to its inhibitory action on cell-cell adhesion. *In silico* characterization of purpureomaculin sequence predicted a stable, hydrophilic small polypeptide molecule, with features that may favorably unleash its pharmaceutical potential in the development of a peptide-based anticancer therapeutic. Nevertheless, further studies are required to investigate the peptide delivery system and immunogenicity of the peptide, which are the main obstacles of most current peptide-based therapeutics. 

### Abbreviations

5-FU: 5-fluorouracil; ACN: acetonitrile; ATCC: American Type Culture Collection; DMEM: Dulbecco’s modified Eagle’s medium; DMSO: dimethyl sulfoxide; DTT: dithiothreitol; EMEM: Eagle’s minimum essential médium; FBS: fetal bovine serum; IAA: iodoacetamide; IC_50_: median inhibition concentration; LAAO: L-amino acid oxidase; MEGM: mammary epithelial cell growth medium; MTP: Malaysian *T. purpureomaculatus*; pI: isoelectric point; RP-HPLC: reversed-phase high performance liquid chromatography; SI: selectivity index; SPI: score peak intensity; TFA: trifluoroacetic acid.

## Supplementary material

Additional file 1. LC-MS/MS of purpureomaculin isolated from Malaysian *Trimeresurus purpureomaculatus* venom (Fraction 1)
